# Exploring the Personality Characteristics of Rhinoplasty Patients: Perfectionism, Rumination, and Self-Compassion

**DOI:** 10.1007/s00266-024-04019-9

**Published:** 2024-05-28

**Authors:** Utku Mete, Uygar Levent Demir, Rümeysa Ayşe Güllülü, Salih Saygin Eker, Fatih Duman, Nadir Altun

**Affiliations:** 1 Department of Otolaryngology-Head and Neck Surgery, Bursa Uludag University Hospital, Görükle Center Campus, Nilüfer, 16059 Bursa, Turkey; 2 Department of Psychiatry, Bursa Uludag University Hospital, Görükle Center Campus, Nilüfer, 16059 Bursa, Turkey

**Keywords:** Aesthetic, Perfectionism, Personality, Rhinoplasty, Rumination, Self-compassion

## Abstract

**Objective:**

This study investigates differences in personality characteristics, including perfectionism, ruminative thinking style, and self-compassion, between individuals who have undergone rhinoplasty and a control group without any history of cosmetic surgery.

**Methods:**

The study included 33 adult patients who underwent rhinoplasty between 2021 and 2023 at Bursa Uludağ University Faculty of Medicine Hospital and 33 adult patients who visited our centre for other complaints as a control group. The rhinoplasty group consisted of primary surgical patients with functional and cosmetic concerns, excluding those who sought revision surgery or had only functional problems. The control group consisted of individuals with no prior cosmetic surgery history and no expectations of aesthetic interventions. Psychiatric analysis was performed using Frost multidimensional perfectionism scale, ruminative thinking style questionnaire, and self-compassion scale.

**Results:**

This research revealed that individuals who had rhinoplasty scored higher in perfectionism 109.3 (±23.3) and ruminative thinking 87.9 (±22) compared to those who did not undergo surgery 94.15 (±22.2) and 77.7 (±23), respectively. Additionally, the rhinoplasty group had lower self-compassion scores, 80.4 (±17.3), than the control group, 86.1 (±11.2). Statistically significant differences were observed in perfectionism between the groups (*p* = 0.009). In rhinoplasty patients, a notably positive correlation was found between perfectionism and ruminative thinking scores (*r* = 0.482; *p* = 0.005), while a moderately significant negative correlation was observed between self-compassion and ruminative thinking scores (*r* = − 0.465; *p* = 0.006).

**Conclusion:**

Individuals who undergo rhinoplasty generally show increased levels of perfectionism and are more prone to ruminative thinking. They also demonstrate reduced self-compassion compared to non-surgical control groups. Cosmetic surgeons should be aware of these psychological trends and consider using appropriate scales during pre-surgery consultations and follow-up visits. Adopting this informed approach can improve the surgeon–patient relationship and help overcome communication challenges.

**Level of Evidence III:**

This journal requires that authors assign a level of evidence to each article. For a full description of these Evidence-Based Medicine ratings, please refer to the Table of Contents or the online Instructions to Authors www.springer.com/00266.

**Supplementary Information:**

The online version contains supplementary material available at 10.1007/s00266-024-04019-9.

## Introduction

Cosmetic surgical procedures have continued to gain increasing popularity over the years [1]. According to the latest American Society of Plastic Surgeons report, cosmetic surgery procedures have grown by 19%, comparing the 2022 Procedural Statistics to 2019 [2]. Like worldwide trends, rhinoplasty has recently been on the rise in our country and has become one of the most frequently performed facial plastic surgery procedures.

The face is a crucial aspect of our body, serving various functional and sensory purposes. It plays a vital role in our interactions with the external world, reflecting emotional changes and conveying the general appearance of an individual's feelings [3]. The nose is a centrally located structure in the face that plays a critical and significant role in facial aesthetics.

Perfectionism is the tendency for an individual to demand a level of performance quality that exceeds the ordinary, either from themselves or from the events in their surroundings. Perfectionism provides support among individuals and seeks to establish a better self-image for the person [4, 5].

Rumination refers to the state of the mind being preoccupied with a single thought or subject, which can manifest in various mental health conditions. It is characterized by an uncontrolled and intrusive nature, displaying repetitive patterns [6]*.*

Self-compassion, a terminology introduced in recent years, is characterized by individuals who display a gentler, kinder, and more selfless attitude towards life, their surroundings, and themselves when facing challenges [7]. Self-compassion provides more emotional resilience and stability than self-confidence, and it involves self-evaluation as much as self-worth, without ego defences and self-aggrandizement [8].

During cosmetic surgery consultations, patients often seek to improve their visual appearance to gain self and social acceptance. This idea of desire has given us an essential foundation for investigating perfectionism. Such patients tend to exhibit persistent concerns and repetitive questions about their appearance before and after the surgery, indicating a tendency towards ruminative thinking patterns. The decision to have cosmetic surgery can be impacted by an individual's ability to accept themselves as they are. It is essential to cope with any emotional and physical challenges that may arise after the procedure and to accept the outcome. These concerns have led researchers to investigate the idea of self-compassion.

This study aims to identify unexplored personality traits, such as perfectionism, rumination, and self-compassion, in both rhinoplasty patients and controls. It also examines how these characteristics interrelate in individuals who undergo rhinoplasty.

## Materials and Methods

### Ethics and Informed Consent

This study received approval from the Faculty of Medicine Clinical Research Ethics Committee, with the decision dated 11 October 2023 and numbered 2023-19/43. Informed consent forms were obtained from all participants after providing them with information about the study's content and purpose.

### Patient Selection

We studied 33 adult patients who underwent rhinoplasty surgery from 2021 to 2023 at our university hospital. Additionally, we included a control group of 33 people who visited our centre for various reasons.

The selection criteria for participants in the rhinoplasty group were individuals who underwent surgery to enhance their aesthetics. We excluded patients who underwent nasal airway procedures based solely on functional considerations (such as septal reconstructions and nasal valve surgeries) and revision operations.

The control group was chosen to be as homogeneous as possible to demonstrate the difference in personality traits with the rhinoplasty group. We preferred to recruit volunteers from individuals who presented with various complaints to the otolaryngology clinic of our university hospital. The control group was formed to have demographic characteristics similar to those of the rhinoplasty group, including being Caucasian and having a similar age and gender range. To be included in the control group, individuals were required to meet certain criteria, such as not having undergone any surgical or office procedures to improve the appearance of any part of their body before and having never felt the need for such interventions. Individuals with a history of cosmetic surgery, injections of fillers and botulinum toxins, or similar local interventions, or those who expressed intentions to undergo such procedures in the future, were not included in the control group since the psychological dimensions of rhinoplasty were examined.

Both the patient and control groups excluded individuals with a history of psychiatric medication use and psychiatric inpatient admissions.

### Data Collection

Demographic data of the participants were collected by a specialist in the department of otorhinolaryngology, while a specialist in the department of psychiatry acquired records related to psychological communication and analysis. The data were collected through face-to-face interviews, and the psychiatric examination involved using native language versions of the self-compassion scale, ruminative thought style questionnaire, and Frost multidimensional perfectionism scale.

### Psychiatric Scales for Personality Characteristics

#### Frost Multidimensional Perfectionism Scale

The tool designed for a multidimensional assessment of perfectionism consists of 35 items. The scale includes six sub-dimensions, and each item is rated on a Likert-type scale ranging from 1 (strongly disagree) to 5 (strongly agree), with a potential score ranging from a minimum of 35 to a maximum of 165 points [9]*.* Psychometric properties of the native language version of the scale were researched.

#### Ruminative Thought Style Questionnaire

This scale comprises 20 items and is organized based on a seven-point Likert-type scoring system [6]. Participants assess the extent to which the statements reflect their own experiences on a scale from 1 (not describing me at all) to 7 (describing me very well), with a potential score ranging from a minimum of 20 to a maximum of 140*.* The adaptation of the scale into the native language and the validation–reliability studies have been conducted and used in the survey.

#### Self-Compassion Scale

The initial form of this scale comprises 26 items distributed across six sub-scales [10]. Respondents must indicate the frequency with which they exhibit behaviours associated with the given scenarios by utilizing a five-point Likert scale, where responses range from 1 (almost never) to 5 (almost always), with a total score ranging from a minimum of 26 to a maximum of 130. This scale follows a single-factor structure, with questions numbered 1, 3, 5, 7, 10, 12, 15, 17, 19, 22, and 23 scored in reverse order (1 = 5, 2 = 4, 3 = 3, 4 = 2, 5 = 1). The scale used in this study was adapted to the native language.

There are no specific cut-off scores for scales. The scores obtained from each test indicated the extent to which patients deviated from or adhered to the examined personality traits.

### Statistical Analysis

The distribution of the data was assessed using the Shapiro–Wilk test. Descriptive statistics for quantitative data were expressed as mean and standard deviation or median (minimum–maximum), while for qualitative data, they were presented as frequency and percentage. In the case of data exhibiting a normal distribution, the t-test was employed to compare two groups. In contrast, the Mann–Whitney U test was utilized when normal distribution was not observed. Pearson’s Chi-square, Fisher–Freeman–Halton, and Fisher’s exact Chi-square tests were applied to analyse categorical data. Relationships between variables were examined using the Pearson correlation coefficient. A significance level of *α* = 0.05 was set. Statistical data analysis was performed using IBM SPSS 28.0 (IBM Corp. Released 2021. IBM SPSS Statistics for Windows, Version 28.0. Armonk, NY: IBM Corp.) statistical software package.

## Results

### Demographic Outcomes and Analyses Between Groups

The mean age in the rhinoplasty group was 29.2 (±9.3) years, compared to 33.5 (±11) years in the control group. The rhinoplasty group included 17 males (51.5%) and 16 females (48.5%), whereas the control group had 15 males (45.5%) and 18 females (54.5%). No statistical differences in age or gender were observed between the rhinoplasty and control groups (Table 1).

**Table 1:** Demographic comparison between rhinoplasty and control groups

	Rhinoplasty group	Control group	*p-*value
Age (years)	29.2 (± 9.3)	33.5 (± 11)	*p = 0.114* (Mann–Whitney U test)
Gender	Male 17 (51.5%) Female 16 (48.5%)	Male 15 (45.5%) Female 18 (54.5%)	*p = 0.805* (Chi-Square test)

### Comparison of Demographic Data Between Perfectionism, Rumination, and Self-Compassion

We examined the demographic data of the patients who participated in the study and compared it to their personality traits. Our analysis revealed no significant correlation between age and gender and the personality traits of perfectionism, rumination, and self-compassion (Table 2).

**Table 2:** Analysis of personality traits by demographic categories

Demographic categories	Perfectionism	Rumination	Self-compassion
Age (ANOVA)	F= 0.236 *p = 0.871*	F= 0.988 *p = 0.404*	F= 0.096 *p = 0.962*
Gender (t-test)	t= 0.576 *p = 0.566*	t= -1.631 *p = 0.108*	t= -0.229 *p = 0.820*

### Comparison of Personality Traits in Rhinoplasty and Control Groups

**Fig. 1:**
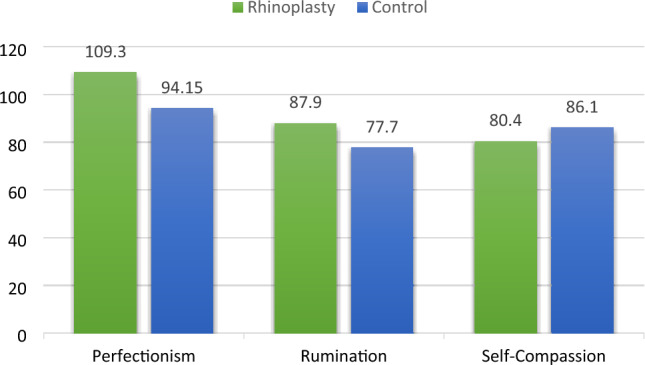
Perfectionism, ruminative thinking style, and self-compassion scores in rhinoplasty and control groups

The study revealed notable distribution between individuals who underwent rhinoplasty and those in the control group. Participants in the rhinoplasty group exhibited higher scores in perfectionism 109.3 (±23.3) and ruminative thinking 87.9 (±22) compared to their counterparts who did not undergo surgery 94.15 (±22.2) and 77.7 (±23), respectively. Additionally, the rhinoplasty group demonstrated lower scores in self-compassion, 80.4 (±17.3), in contrast to the control group, 86.1 (±11.2) (Fig. 1).

The statistical analyses revealed significant differences between the groups regarding perfectionism (*p* = 0.009). While the distinctions in ruminative thinking (*p* = 0.071) and self-compassion (*p* = 0.122) approached noteworthy levels, they did not reach statistical significance.

### Exploration of Personality Traits Across Males and Females

We analysed the data to investigate gender differences in perfectionism, ruminative thinking style, and self-compassion scores. The male participants had a high perfectionism score of 102.8 (±20.2), while their ruminative thinking and self-compassion scores were 78.1 (±20.7) and 82.8 (±14.2), respectively. On the other hand, the female participants had a slightly lower perfectionism score of 99.50 (±26), but a higher ruminative thinking score of 87.2 (±24.3). However, their self-compassion scores were similar to males, averaging 83.6 (±15.5). Male and female participants had no statistical difference in studied personality characteristics (*p* = 0.566, *p* = 0.108, and *p* = 0.820, respectively).

### Interactions Between Personality Scales in Rhinoplasty Group

**Fig. 2:**
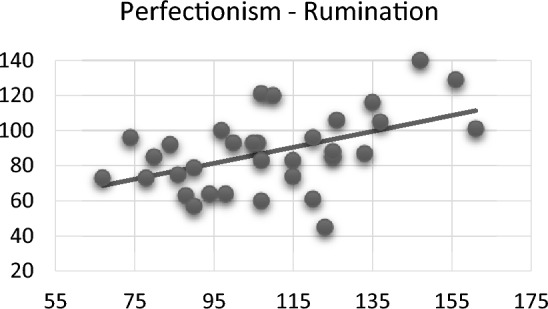
Correlation between perfectionism and rumination

**Fig. 3:**
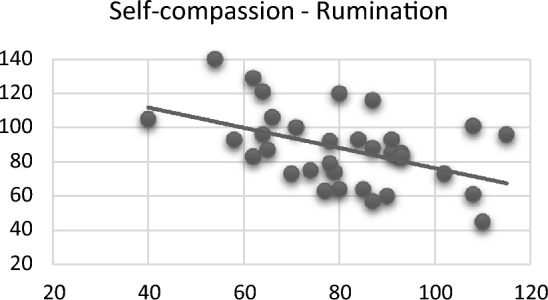
Correlation between self-compassion and rumination

When examining the relationship between psychiatric scales in rhinoplasty patients, a relatively meaningful positive relationship was found between perfectionism and ruminative thinking scores (*r* = 0.482; *p* = 0.005) (Fig. 2), while a moderately significant inverse relationship was found between self-compassion and ruminative thinking scores (*r* = − 0.465; *p* = 0.006) (Fig. 3).

## Discussion

In the past literature, personality traits like dependency, histrionic behaviour, and narcissism have been studied in patients who undergo aesthetic surgery [11]. Additionally, studies have explored and emphasized the importance of borderline personality and body dysmorphic disorder in suitability for cosmetic interventions [12]. Patients undergoing aesthetic surgeries often exhibit various levels of symptoms in body dysmorphic disorder [13]. However, our focus is on studying personality traits that have not yet been investigated.

Perfectionism has been conceptualized as a pervasive and demanding trait, broadly defined as having excessively high standards. It has been linked to a range of psychological and physical disorders like depression, obsessive–compulsive personality disorder, erectile dysfunction, irritable bowel syndrome, and migraine [9, 14]. We believe that high expectations regarding the outcomes of rhinoplasty, particularly the appearance of the nose and facial profile, may be closely associated with a perfectionist personality trait.

There are two types of perfectionism: normal perfectionism involves setting high standards for oneself and being unforgiving of one's mistakes. In contrast, neurotic perfectionism involves an unrelenting pursuit of perfection and a refusal to be satisfied with one's accomplishments [15]. In the case of neurotic perfectionism, the focus is on fearing mistakes rather than achieving success. This fear of making errors is associated with a dichotomous thinking style, which is interpreted as being linked to depression [16].

Another aspect of perfectionism is reflected in the literature on obsessional experiences, where the reluctance to complete a task is described as an uncertain state of doubt, categorized perfectionists based on this "reluctance to complete a task" observed in individuals with obsessive–compulsive tendencies [17]. The parental dimension of perfectionism has been deliberated, suggesting that it develops in individuals raised in families where the emphasis is placed on receiving love and approval based on one's achievements. Lastly, epitomized by the phrase "there is a place for everything, and everything must be in its place", perfectionists exhibit orderliness and meticulousness [9].

In our findings, we observe traits of perfectionism in rhinoplasty patients, consistent with Babuccu et al.'s prior study, which also investigated various personality traits across different genders. It was noted that female patients displayed characteristics like impulsiveness, childlike tendencies, egocentrism, and self-oriented perfectionism. In contrast, male patients were more inclined to exhibit rigidity, stubbornness, and suspicion and demonstrated perfectionism [18].

Perfectionists often tend towards obsessive rumination and repetitive cognitive responses to stress, termed perseverating perfectionism. Perfectionism manifests in two forms based on its origin: self-oriented perfectionism (SOP) and socially prescribed perfectionism (SPP). SOP refers to high standards set by oneself, while SPP pertains to standards imposed by societal expectations. Both SOP and SPP are linked to cognitive perseveration [19]. Another study supports the idea of perfectionism cognition theory that perfectionists are more likely to suffer from interrelated forms of cognitive perseveration, and they may experience emotional distress due to the positive interactions between SOP and SPP with worry and rumination [20]. Our study found a positive correlation between perfectionism and ruminative thoughts in the rhinoplasty group. We hypothesize that socially imposed high standards of perfectionism may be a driving factor for individuals seeking rhinoplasty, as it intensifies a ruminative thought process to achieve an aesthetically perfect appearance.

Humans are thought to be the only species capable of self-reflection. Ruminative thinking is crucial to this ability, involving repetitive, recurrent, intrusive, and uncontrollable thoughts about a particular subject or situation [6]. There is also a link between the style of ruminative thinking and depression, which is one of the three core criteria outlined in the response styles theory, which is designed to examine the relationship between these two concepts [21]. Moreover, rumination has been shown to exacerbate negative thought patterns, decrease problem-solving skills, disrupt instrumental behaviour, and reduce social support. Additionally, there is evidence suggesting that rumination is linked to various psychopathologies, including anxiety, binge eating, and self-harm [22].

Self-compassion refers to individuals' ability to treat themselves with kindness and empathy during challenging times, such as experiencing pain or failure. This includes seeing negative experiences as a natural part of life and finding logical solutions instead of dwelling on negative thoughts and emotions [23]. Understanding one’s thoughts and emotions and those of others is integral to the concept of self-compassion and the theory of mind. This concept is closely related to other cognitive constructs, such as self-concept, mindfulness, spirituality, common humanity, kindness, and empathy [24].

It is essential to distinguish between two key concepts: self-esteem and self-compassion. Studies have shown that self-compassion is a stronger predictor of stable self-worth than self-esteem and less reliant on specific outcomes. Additionally, self-compassion is associated with lower levels of social comparison, public self-consciousness, rumination, anger, and the need for cognitive closure. On the other hand, self-esteem is positively linked to narcissism, unlike self-compassion [25]. In our study, we identified a reverse correlation relationship, indicating that as self-compassion increases, there is a decrease in rumination, aligning with this knowledge.

While this study provides valuable insights, it is limited by its sample size and the specific demographic of the participants, primarily those seeking rhinoplasty for aesthetic and functional purposes at a tertiary university hospital. A study examining patients seeking aesthetic or functional rhinoplasty revealed a greater presence of psychopathology and depression in those undergoing aesthetic rhinoplasty^26,27^. It is important to note that the outcomes may differ when comparing our data with studies conducted on patient groups undergoing surgery solely for cosmetic or functional purposes. Future research could expand on these findings by including a broader and more diverse demographic and comparing individuals seeking purely cosmetic versus functional rhinoplasty.

In preparation for cosmetic surgery procedures, the physician and the patient should need to establish a shared understanding of the goals and expectations. The surgeon should focus on enhancing the physical appearance and closely monitor the patient's psychological and personality traits. To identify any potential issues that may arise during the surgery or recovery, the physician can administer surveys such as Frost multidimensional perfectionism scale, ruminative thought style questionnaire, and self-compassion scale to assess the patient’s personality traits, such as perfectionism, ruminative thinking style, and self-compassion. These traits can also be indicative of underlying psychiatric disorders. If any red flags are raised, the surgeon may seek consultation from the psychiatric unit or reconsider the patient’s eligibility for cosmetic surgery. It is essential to consider the patient’s mental and emotional well-being and physical appearance.

The study provides valuable information on the psychological characteristics of individuals who undergo rhinoplasty. This information highlights the significance of considering psychological factors when assessing patients before surgery. By understanding these personality traits, surgeons and mental health professionals can better prepare and support patients, potentially enhancing their overall psychological well-being and satisfaction after the surgery.

## Conclusion

The study revealed that rhinoplasty patients exhibit higher perfectionism levels than the control group. Additionally, a positive correlation was discovered between perfectionism and ruminative thinking, while a negative interaction was presented between self-compassion and ruminative thinking in rhinoplasty patients. The complex relationship among perfectionism, ruminative thinking, and self-compassion in these patients offers an intriguing subject for further research. Our results shed light on the particular personality characteristics of aesthetic surgery patients, especially from the rhinoplasty perspective.

## Supplementary Information

Supplementary file 1 (DOCX 292 kb)
