# Supplementary material for: Exploring the Personality Characteristics of Rhinoplasty Patients: Perfectionism, Rumination, and Self-Compassion
Source: Aesthetic Plast Surg. 2024 May 28;48(24):5299–305. doi: 10.1007/s00266-024-04019-9 (PMC11750891; doi:10.1007/s00266-024-04019-9)

6. SUPPLEMENTARY MATERİAL

6.1. Frost Multidimensional Perfectionism Scale


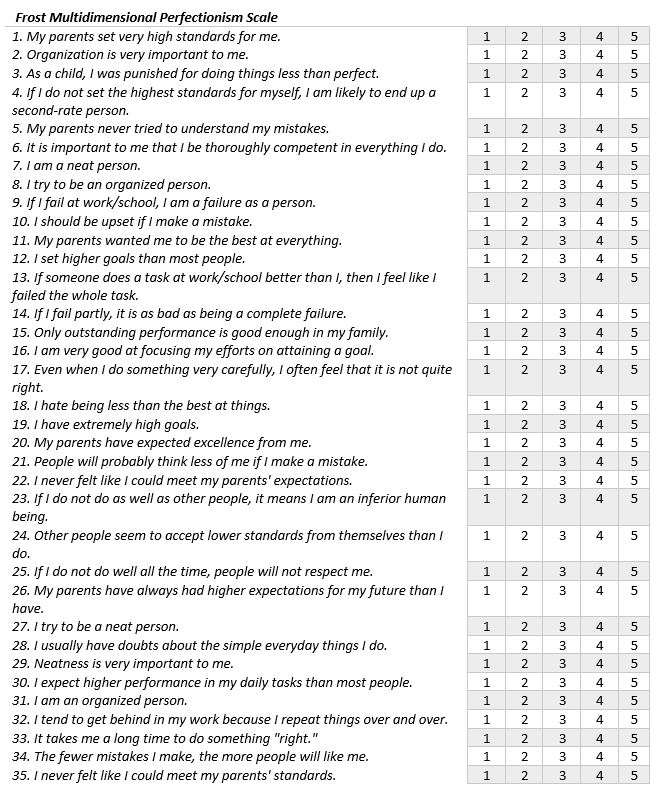


6.2. Ruminative Thought Style Questionnaire


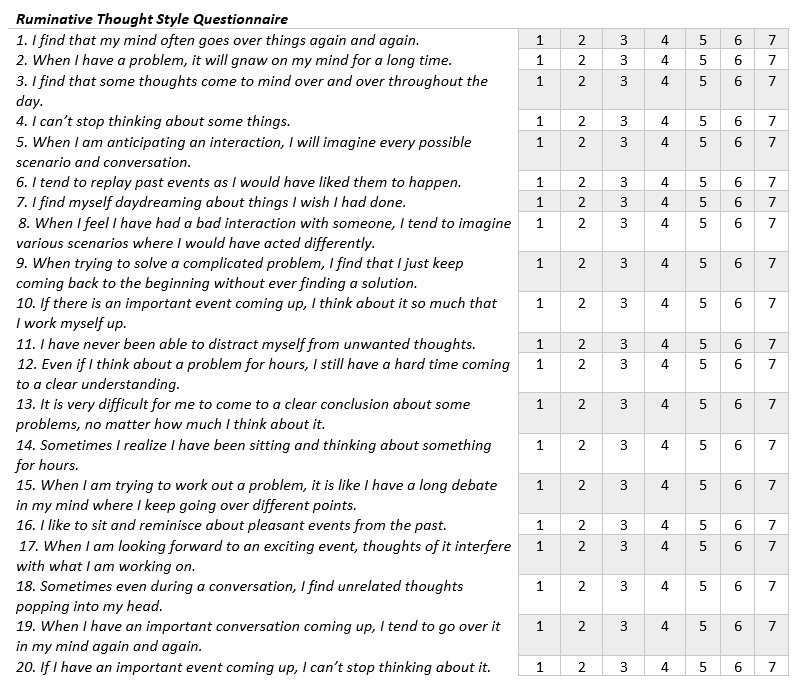


6.3. Self-Compassion Scale


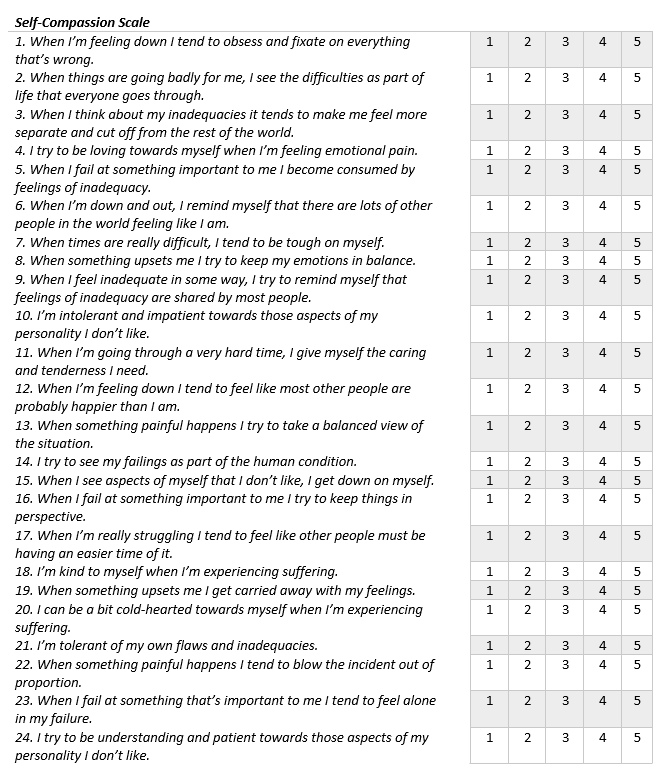

Supplement: Supplementary file 1 — (DOCX 292 kb) [file 266_2024_4019_MOESM1_ESM.docx]
